# Melatonin Improves Intestinal Barrier Impairment in a Mouse Model of Autism Spectrum Disorder

**DOI:** 10.3390/biology14111594

**Published:** 2025-11-14

**Authors:** Francesca Sulas, Gaia Favero, Sara Anna Bonini, Claudio Lonati, Daniela Pinto, Maurizio Memo, Fabio Rinaldi, Rita Rezzani

**Affiliations:** 1 Anatomy and Physiopathology Division, Department of Clinical and Experimental Sciences, University of Brescia, 25123 Brescia, Italy; francesca.sulas@unibs.it (F.S.); gaia.favero@unibs.it (G.F.); claudio.lonati@unibs.it (C.L.); 2 Interdepartmental University Center of Research Adaption and Regeneration of Tissues and Organs (ARTO), University of Brescia, 25123 Brescia, Italy; 3 Department of Molecular and Translational Medicine, Division of Pharmacology, University of Brescia, 25123 Brescia, Italy; sara.bonini@unibs.it (S.A.B.); maurizio.memo@unibs.it (M.M.); 4 Italian Society for the Study of Orofacial Pain (Società Italiana Studio Dolore Orofacciale—SISDO), 25123 Brescia, Italy; 5 Human Microbiome Advanced Project Institute, 20129 Milan, Italy; dpinto@giulianipharma.com (D.P.); fabio.rinaldi@studiorinaldi.com (F.R.)

**Keywords:** autism spectrum disorder, gut, BTBR mice, tight junctions, melatonin

## Abstract

Autism spectrum disorder (ASD) is a neurodevelopmental condition mainly characterized by social impairments and repetitive behaviors. An altered intestinal barrier morphology and increased transmucosal leaks have also been implicated in ASD; in fact, comorbidities such as gastrointestinal problems (leaky gut) have frequently been reported in these patients. The regulation of tight junctions (TJs) is essential in maintaining intestinal barrier morphology and in regulating the delicate balance of trafficking between the intestinal lumen and the submucosa. To date, there are no definitive treatments for ASD comorbidities; however, melatonin (MLT) represents a well-validated and tolerated treatment for sleep disorders in ASD patients. The potential beneficial effects of MLT on this disorder have been and continue to be better investigated. In this context, the present study examines the effects of oral MLT administration (10 mg/kg/day for 16 weeks) on the intestinal barrier in BTBR T + Itpr3tf/J (BTBR) mice, a validated ASD model. Morphological analyses of the ileum of these animals reveal modified villus height (Vh), crypt depth (Cd), and Vh–Cd ratios; an inflammatory state; and a decrease in Paneth cells. Moreover, these mice showed altered TJ expression compared to the control animals (C57BL6/J mice). Notably, MLT normalizes morphological indices and TJ expression, consistent with an improved gut barrier morphology. These data collectively suggest that orally administered MLT can promote the remodeling of the intestinal barrier; thus, we can suppose that MLT reduces gastrointestinal barrier leaks. The overall safety and economy of MLT use suggest that this indolamine could be efficacious as an adjuvant therapy to reduce the condition known as leaky gut.

## Simple Summary

Many individuals with autism spectrum disorder experience not only challenges with social interaction and behavior but also gastrointestinal problems such as dysbiosis and increased gut permeability. These issues may be linked to changes in the gut barrier, which normally acts to restrict the passage of ions, molecules, and cells through the paracellular space. In this study, we explored whether melatonin, an endogenous biomolecule often used in sleep disorders, could also help improve gut health in a well-established mouse model of autism. Mice with autism-like traits showed altered intestinal villi, signs of gut inflammation, and impaired gut barrier function. After being treated with melatonin every day for 16 weeks, these mice showed improvements in gut structure and reduced inflammation. Most importantly, the integrity of the gut barrier improved, likely due to the modulation of key proteins that control its function, which may help prevent harmful substances from entering the body. These results suggest that melatonin could improve gut barrier integrity and overall well-being, offering a new perspective on managing autism-related comorbidities.

## 1. Introduction

Autism spectrum disorder (ASD) is a group of neurodevelopmental disorders characterized by impairments in communication, social interest, and stereotypical behavior. This disorder primarily affects children, as reported in several clinical papers [1,2]. Many efforts have been made to evaluate the symptoms of these disorders, but their etiology and manifestations remain unclear [3].

More recently, gastrointestinal (GI) abnormalities have been highlighted in several patients with ASD; these alterations involve the intestinal morphology and therefore also have repercussions on gut microbiota [4].

Clinical and experimental studies have underlined an alternate intestinal barrier morphology in ASD (leaky gut) and demonstrated that increased intestinal permeability is associated with the disruption of tight junctions (TJs). This has led to the entry of ions, solutes, and various immune cells, resulting in local inflammation and cellular oxidative stress [5,6,7]. TJs are multi-protein complexes located at the apical region of the lateral membrane between adjacent intestinal epithelial cells. They play a central role in regulating the intestinal barrier function by sealing the paracellular space, thus controlling the selective permeability to ions, solutes, and immune mediators, and maintaining epithelial morphology and polarity [8].

Under physiological conditions, the integrity of the intestinal barrier is maintained by various TJs, such as claudins, occludins, and zonulins [4,9,10,11,12], key components involved in the dynamic equilibrium with both intracellular and extracellular events [13]. In particular, zonula occludens-1 (ZO-1), also known as TJ protein 1 (TJP-1), is a membrane-associated protein that ensures the basolateral cell–cell adherence in intestinal epithelial cells by cross-linking other transmembrane TJs (e.g., claudins, occludins, and junction adhesion molecules) to the actin cytoskeleton [14,15,16]. ZO-1 has been shown to regulate the expression of intestinal TJs, modulate the inflammatory state, and control the transmigration of immune cells from the gut to the bloodstream [17]. More recently, it has been shown that the reduction and delocalization of ZO-1 is linked to intestinal morphology, homeostasis, and permeability in aged mice treated with methylglyoxal, which is a precursor implicated in the pathogenesis of type 2 diabetes and neurodegenerative diseases [18]. Furthermore, when ZO-1 is deregulated, both intestinal and extraintestinal autoimmune, inflammatory, and neoplastic disorders occur [19]. In addition, serum zonulin levels are associated with increased intestinal permeability and symptom severity in patients with ASD [20].

Regarding other cell adhesion molecules, several studies suggest that altered expression of claudins affects the permeability of both the blood–brain barrier and the intestinal barrier [2]. Specifically, claudin-1 and occludin are downregulated in the colonic tissue of the valproic acid-induced autism model [4,21]. Conversely, claudin-2 is upregulated in the small and large intestines in several inflammatory diseases, contributing to diarrhea via a leak flux mechanism [22].

The mucosal immune system, including epithelial cells, plays a crucial role in maintaining this equilibrium. Paneth cells (Pcs), a specialized type of epithelial cells found in intestinal crypts, are an important source of antimicrobial peptides. Pcs provide intestinal host defense against pathogens and control the healthy microbiota by secreting antimicrobial peptides, such as α-defensin 5, which presented strong microbicidal activity [23,24]. These cells are the focus of studies investigating homeostasis mechanisms in the GI tract and their collapse in infection and chronic inflammation processes [25].

Interventions targeting the gut “ecosystem”, through diet modification, probiotics, symbiotics, or antioxidants, offer therapeutic promise. These adjunctive treatments, compared with existing treatment methods, may improve the quality of life of ASD patients [26]. However, rigorous and large-scale studies are needed to clarify mechanisms of action, long-term efficacy, and safety.

To address this question, in the present study, we treated BTBR T + Itpr3tf/J (BTBR) mice, a well-established model for ASD assessment [27,28], with melatonin (N-Acetyl-5-methoxytryptamine, MLT), which is an indolamine produced by the pineal gland and several extra-pineal sources, including the GI tract [29,30]. MLT is well known for its role in the regulation of the human circadian rhythm [31] and is commonly used as a supplement to manage sleep-related conditions such as insomnia, anxiety, and jet lag [32]. Furthermore, MLT is a versatile and pleiotropic molecule with antioxidant, anti-tumor, and anti-inflammatory effects across various systems and cancer types [33,34,35,36]. Recent studies have shown that MLT exerts beneficial effects on the digestive system, including improved paracellular permeability in the duodenal mucosa and enhanced bicarbonate secretion [37,38]. Reduced MLT secretion has been observed in individuals with ASD, and its administration is a well-established and well-tolerated treatment for sleep disorders in children and adolescents with ASD. In addition to regulating sleep, we evaluated the potential MLT effects in alleviating intestinal barrier morphological alterations correlated to ASD comorbidities.

BTBR mice exhibit multiple behavioral phenotypes relevant to all core diagnostic symptoms of autism. BTBR mice display reduced social approach, limited reciprocal social interactions, impaired juvenile play, and excessive self-grooming compared to C57BL/6J controls [28].

The overall goals of this study were to assess whether intestinal barrier morphology and integrity could contribute pathophysiologically to ASD and to identify a possible perspective in ASD therapy, considering the involvement of the GI tract in this disorder. Thus, melatonin is emerging as a central node in adjunctive treatments against gut dysbiosis.

## 2. Materials and Methods

### 2.1. Animal Experimental Groups

The experimental groups were established in accordance with previous studies of our research group [39]. A total of 20 male BTBR T + Itpr3tf/J (BTBR) mice (JAX™ Mice Strain; The Jackson Laboratory, Bar Harbor, ME, USA), used as a transgenic animal model of ASD, and 20 male C57BL6/J mice (JAX™ Mice Strain; The Jackson Laboratory, Bar Harbor, ME, USA), serving as healthy controls (CTRs), were housed in rodent polysulfone home cages (2–3 animals per cage—369 × 165 × 132 mm—1145 T) under standardized conditions and 12/12 h dark/light cycles to minimize circadian variability. The cages were equipped with woodchips for bedding and nesting materials, and food and water were provided ad libitum, in accordance with the 2010/63/EU directive. Temperature (22 °C) and humidity (50 ± 10%) in the cage were automatically regulated by the Sealsafe Aero System through individually ventilated cages with EPA filters (Tecniplast Group, Buguggiate (VA), Italy).

The composition and macronutrients of the rodent chow are summarized in Table 1. All efforts were made to minimize animal suffering and the number of animals used. All experimental procedures were approved by the Italian Ministry of Health (n° 446/2018-PR–20 June 2018) and performed according to European Communities Council Directive guidelines (CEE N°86/609).

The animals were trained for gavage during the first four weeks of life using NaCl 75 mM solution. The experiment started at the beginning of the sixth week of life and finished at the end of the twenty-first week of life, for a total of sixteen weeks of treatment.

Each experimental group (BTBR and CTR) was divided into two subgroups by a researcher blinded to the experimental protocol, resulting in four experimental subgroups of 10 mice each. Randomly, one BTBR and one CTR subgroup received 10 mg/kg/day of MLT for 16 weeks via oral gavage, while the remaining subgroups received the vehicle (1% ethanol), as described by Borsani et al. [40]. Briefly, MLT was administered orally in a single daily dose through a 100 μL gavage at approximately 6:30 p.m., aiming to mimic the physiological pattern and relative circadian rhythm of MLT. The MLT dose administered in this study was chosen based on previous studies [40,41,42,43]. The half-life of MLT was previously reported by Andersen et al. [44]. Body weight was monitored and evaluated using an analytical balance during the experimental period for each animal.

At the end of the treatments, all the experimental animals were subjected to behavioral (self-grooming) tests [40].

**Figure 1:**
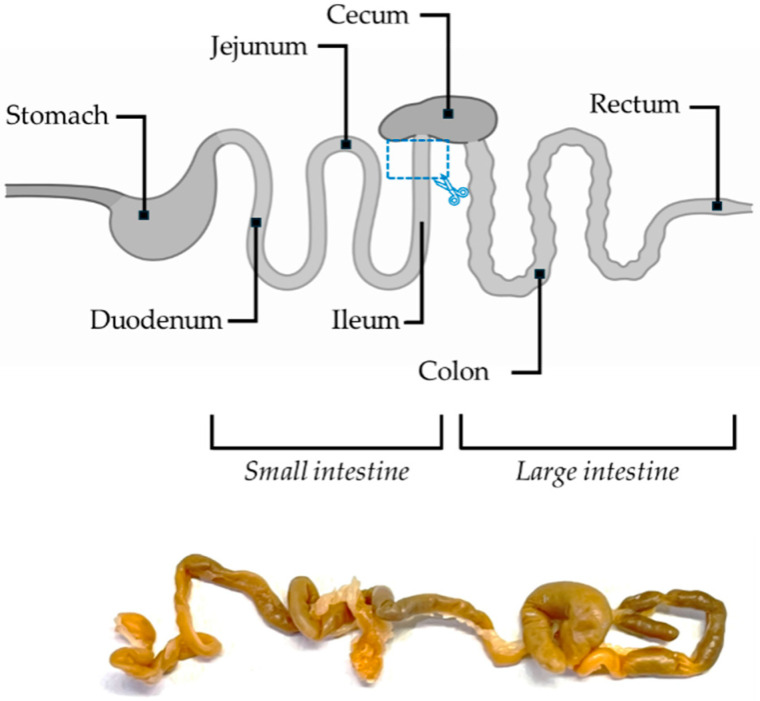
Schematic illustration and real image of the mouse intestine. The site of sample collection is marked by a blue rectangle.

At the end of the treatments, five animals for each experimental subgroup were deeply anesthetized with 5% isoflurane, and all mice were perfused transcardially with saline, followed by 50 mL of 4% paraformaldehyde in 0.1 M phosphate-buffered saline. The gut was then removed for morphological and immunohistochemical analysis. Specifically, the ileum (part of the small intestine) was isolated (Figure 1), rinsed in physiological saline, and incubated for 24 h in 4% paraformaldehyde (PFA) in 0.01 M phosphate-buffered saline (PBS). Samples were then washed twice with 0.01 M PBS for 10 min each and rinsed in 50% ethanol for 2 h at room temperature. Following this step, the samples were immersed in 70% ethanol for at least one week at 4 °C.

Before paraffin embedding, the samples were immersed in 95% ethanol twice for 2 h at room temperature, followed by immersion in 100% ethanol for the same duration. The tissues were then placed in xylene for 10 min until they became transparent. Subsequently, the samples were incubated overnight in a 1:1 mixture of xylene and paraffin at 60 °C, followed by three incubations with pure paraffin, each for 2 h at 60 °C. Finally, the tissues were embedded in paraffin. Serial paraffin sections (5 μm thick) of each sample were cut using a microtome.

The other five animals were cervically dislocated, and the fresh guts were carefully removed for future studies.

### 2.2. Behavioral Tests

Stereotyped and repetitive behaviors are a hallmark of ASD; therefore, animals were observed individually in an arena for 10 min, during which time they were videotaped. The main repetitive behavior most frequently recorded in these experimental groups of mice was self-grooming. The animals were tested during the light phase, from 09:00 a.m. to 04:00 p.m.

Self-grooming tests were performed as previously reported [40,45]. Briefly, each mouse was placed alone into a standard mouse cage. After a 5 min habituation phase, the time spent performing self-grooming was recorded for 10 min with a digital camera (Noldus, Wageningen, The Netherlands, RRID:SCR_004074) and analyzed by the operator, blinded to the genotype and treatment.

### 2.3. Morphological and Morphometrical Evaluations of the Ileum

Alternate sections of the ileum from all experimental animal groups were deparaffinized, rehydrated, and stained with hematoxylin–eosin (Bio Optica, Milan, Italy) using standard procedures. Sections were observed with a light optical microscope (Olympus BX50 Microscope, Hamburg, Germany) at a final magnification of 200× and 400×. Digitally fixed images of all experimental mice were analyzed using a computer-assisted image analysis system (Image Pro-Plus, Milan, Italy) by an examiner blinded to the purpose of the study in order to evaluate the inflammatory state semi-quantitatively and to measure the villus height (Vh) and crypt depth (Cd).

**Figure 2:**
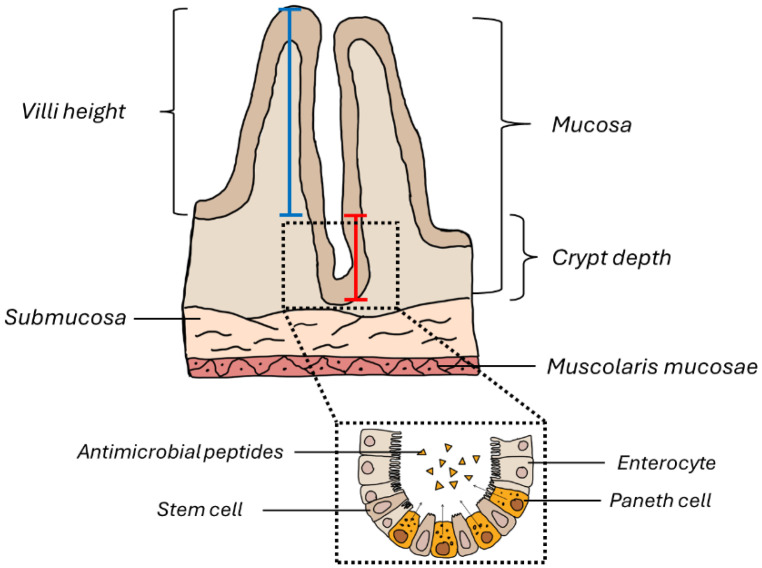
Schematic representation of the ileum wall. The blue line indicates how the morphometric measurement of the intestinal villus height was performed, while the red line indicates how the crypt depth was measured.

Ten random fields for each ileum sample were analyzed to assess the number of Pcs, Vh, Cd, and their ratios. For the evaluation of Vh and Cd, three villi or three crypts were measured per random field. For the analysis of inflammatory cells, ten random fields per group were analyzed; we only considered those in which the entire submucosa was visible. In detail, the inflammatory cells present in the submucosa were evaluated as follows: negative (−), very weak (±), weak (+), moderate (++), and strong (+++). Vh, Cd, and their ratios, along with the assessment of Pc number, were measured in a manner demonstrated in Figure 2.

### 2.4. Immunohistochemical and Immunofluorescence Evaluation of the Ileum

Alternate sections of the ileum from all experimental animal groups were deparaffinized with xylene, rehydrated through a graded ethanol series (100%, 95%, and 70%), and rinsed with distilled water. The sections were then washed in 1× tris-buffered saline (TBS 1×) and Triton 0.3%, and subjected to antigen retrieval in 0.01 M sodium citrate buffer (pH of 6.0; C9999, Sigma-Aldrich™, St. Louis, MO, USA) using a microwave oven for two cycles of 3 min at 600 W. Following this step, the slides were washed in TBS 1× for 5 min and then, for immunohistochemistry analyses only, incubated with 3.5% hydrogen peroxide for 10 min at room temperature. To prevent non-specific binding, for both immunohistochemistry and immunofluorescence, sections were incubated with 3% goat serum (G9023, Sigma-Aldrich™, St. Louis, MO, USA) in TBS 1× for 1 h at room temperature. Next, the sections were incubated for 45 min at 37 °C, followed by 1 h at room temperature with the following primary antibodies (diluted in TBS 1× and 3% goat serum): rabbit polyclonal anti-claudin 1 (SAB4200462, Sigma-Aldrich™, St. Louis, MO, USA) at a 1:100 dilution, rabbit polyclonal anti-claudin 2 (SAB4300737, Sigma-Aldrich™, St. Louis, MO, USA) at a 1:150 dilution, rabbit polyclonal anti-ZO-1 (61-7300, Invitrogen™, Waltham, MA, USA) at a 1:100 dilution, and rabbit polyclonal anti-α-defensin 5 (PAB912Mu02, Cloud-Clone Corp., Katy, TX, USA) at a 1:300 dilution.

For immunohistochemical analyses of claudin-1, claudin-2, and ZO-1, sections were incubated with biotinylated secondary antibodies (BP-9100-50, Vector Laboratories, Newark, CA, USA) for 50 min at room temperature, then with an avidin–biotin peroxidase complex (PK-6100, Vector Laboratories, Newark, CA, USA) for another 50 min at room temperature. Immunoreactivity was visualized using 0.33% hydrogen peroxide and 3,3′-diaminobenzidine (DAB; 11718096001, Roche Applied Bioscience, Basel, Switzerland). Sections were counterstained with Carazzi’s hematoxylin (05-06002, Bio Optica, Milan, Italy), dehydrated, mounted, and examined under a light optical microscope (Olympus BX50, Hamburg, Germany) at final magnifications of 200× and 400×.

For immunofluorescence analyses of α-defensin 5, ileum sections were labeled with Cy3-conjugated anti-mouse antibody (diluted 1:400), counterstained with 4′,6-diamidino-2-phenylindole (DAPI), mounted, and observed with a Zeiss AXIO Observer. Z1 Inverted Fluorescence Microscope (Carl Zeiss, Jena, Germany) with red/green/blue filters at final magnification of 63×.

TBS 1× was used instead of the primary antibody during antibody validation (as negative controls) [46].

The immunostaining intensity was quantified as integrated optical density (IOD) using a computer-assisted image analysis system (Image Pro-Plus, Milan, Italy) and expressed as arbitrary units (AUs). White balancing and background subtraction were applied, followed by the pixel quantification of DAB-positive areas [47,48]. For each animal, ten random fields were analyzed by a blind examiner, and the corresponding IOD values were averaged. Statistical analysis was conducted on these mean values.

### 2.5. Statistical Analysis

Results are expressed as the mean ± standard error of the mean (S.E.M.). Statistical significance among experimental subgroups was assessed using an ordinary one-way ANOVA with Šídák’s multiple-comparison test or a two-way ANOVA with Tukey’s multiple-comparison test, depending on the type of data (GraphPad Prism, Version 9.0). A *p*-value ≤ 0.05 was considered statistically significant.

## 3. Results

All experimental mice remained healthy during the whole experiment, readily consuming their daily food. As previously reported [40,49], body weight growth differed between the two mouse strains. The BTBR mice weighed more than the CTR mice; however, the MLT treatment did not influence the body weight (Figure S1). This is only a baseline evaluation, but it could be considered a starting point for other in-depth analyses to identify a possible correspondence between a clinical variation—body weight—and morphology and immunohistochemistry patterns that characterize the intestinal barrier in ASD models.

### 3.1. Repetitive and Stereotyped Behaviors

After 16 weeks of treatment, mice were tested for repetitive and stereotyped behavioral tasks. Self-grooming is a typical repetitive behavior, so time spent on this was recorded during a 10 min observation phase. The results obtained demonstrate a statistically significant increase in time spent performing self-grooming in BTBR + veh mice compared to CTR + veh mice. No statistically significant differences were observed between CTR + veh and CTR + MLT mice. In contrast, the chronic MLT treatment of BTBR mice was able to improve this negative behavior: indeed, in BTBR + MLT mice, the time spent performing self-grooming was significantly reduced compared to that in BTBR + veh mice. These data suggest a positive effect of MLT in reversing stereotyped and repetitive behaviors, which are core symptoms of ASD (Table S1).

### 3.2. Morphological and Morphometrical Evaluation of the Ileum and α-Defensin 5 Analyses

**Figure 3:**
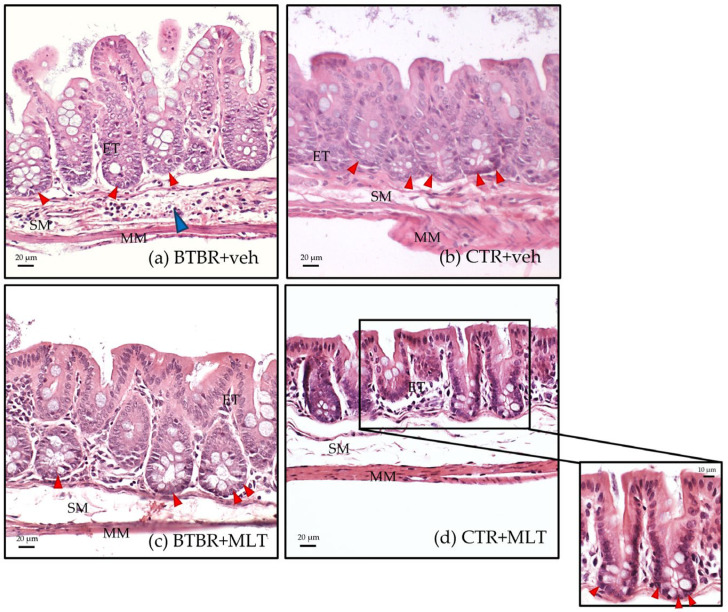
Representative hematoxylin–eosin-stained photomicrographs of ileum samples of BTBR + veh (**a**), CTR + veh (**b**), BTBR + MLT (**c**), and CTR + MLT (**d**) mice. ET: enterocyte; MM: muscularis mucosae; SM: submucosa. The blue arrow indicates inflammatory cell infiltration, while red arrows indicate Paneth cells. Scale bar: 20 µm/10 µm.

**Figure 4:**
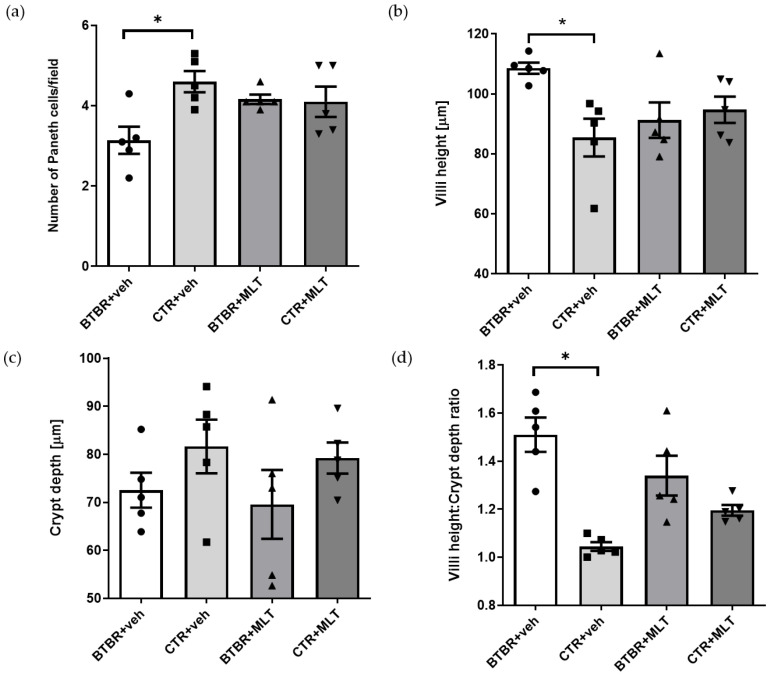
The graphs report, respectively, the results of morphometrical evaluation of Paneth cell number (**a**), villus height (**b**), crypt depth (**c**), and the Vh–Cd ratio (**d**). (**a**) Ordinary one-way ANOVA with Šídák’s multiple-comparison test; F (3; 16) = 4.449; *p*-value = 0.0187. BTBR + veh (*n* = 5) vs. CTR + veh (*n* = 5): mean ± S.E.M. = 3.14 ± 0.339 vs. 4.60 ± 0.265; *p*-value = 0.0081 (*). BTBR + veh (*n* = 5) vs. BTBR + MLT (*n* = 5): mean ± S.E.M. = 3.14 ± 0.339 vs. 4.16 ± 0.117; *p*-value = 0.0731. CTR + veh (*n* = 5) vs. CTR + MLT (*n* = 5): mean ± S.E.M. = 4.60 ± 0.265 vs. 4.10 ± 0.377; *p*-value = 0.5662. (**b**) Ordinary one-way ANOVA with Šídák’s multiple-comparison test; F (3; 16) = 3.970; *p*-value = 0.0272. BTBR + veh (*n* = 5) vs. CTR + veh (*n* = 5): mean ± S.E.M. = 108.5 ± 1.848 vs. 85.42 ± 6.289; *p*-value = 0.013 (*). BTBR + veh (*n* = 5) vs. BTBR + MLT (*n* = 5): mean ± S.E.M. = 108.5 ± 1.848 vs. 91.24 ± 5.916; *p*-value = 0.0718. CTR + veh (*n* = 5) vs. CTR + MLT (*n* = 5): mean ± S.E.M. = 85.42 ± 6.289 vs. 94.71 ± 4.379; *p*-value = 0.4907. (**c**) Ordinary one-way ANOVA with Šídák’s multiple-comparison test; F (3; 16) = 1.189; *p*-value = 0.3454. BTBR + veh (*n* = 5) vs. CTR + veh (*n* = 5): mean ± S.E.M. = 72.53 ± 3.644 vs. 81.62 ± 5.589; *p*-value = 0.5454. BTBR + veh (*n* = 5) vs. BTBR + MLT (*n* = 5): mean ± S.E.M. = 72.53 ± 3.644 vs. 69.56 ± 7.182; *p*-value = 0.9703. CTR + veh (*n* = 5) vs. CTR + MLT (*n* = 5): mean ± S.E.M. = 81.62 ± 5.589 vs. 79.21 ± 3.242; *p*-value = 0.9837. (**d**) Ordinary one-way ANOVA with Šídák’s multiple-comparison test; F (3; 16) = 12.35; *p*-value = 0.0002. BTBR + veh (*n* = 5) vs. CTR + veh (*n* = 5): mean ± S.E.M. = 1.51 ± 0.072 vs. 1.05 ± 0.018; *p*-value < 0.0001. BTBR + veh (*n* = 5) vs. BTBR + MLT (*n* = 5): mean ± S.E.M. = 1.51 ± 0.072 vs. 1.34 ± 0.083; *p*-value = 0.1397. CTR + veh (*n* = 5) vs. CTR + MLT (*n* = 5): mean ± S.E.M. = 1.05 ± 0.018 vs. 1.20 ± 0.022; *p*-value = 0.2177.

BTBR + veh mice had a higher number of inflammatory cells in the submucosa of the ileum (Table 2 and Figure 3a) compared to CTR + veh and CTR + MLT mice (Table 2 and Figure 3b,d). In addition, BTBR + veh mice showed a decrease in the number of Pcs in the crypts (Figure 4a) along with an increase in intestinal Vh (Figure 4b), a reduction in Cd (Figure 4c), and a higher Vh–Cd ratio (Figure 4d) compared to CTR + veh mice. In contrast, CTR + veh and CTR + MLT mice showed “normal” ileum morphology with minimal inflammation, increased Pcs, normal Vh and Cd, and, thus, a lower Vh–Cd ratio (Table 2 and Figure 3b and Figure 4a–d).

Interestingly, treating BTBR mice with MLT reduced the inflammation in the intestinal submucosa (Table 2) and slightly increased the number of Pcs in the crypts (Figure 3c and Figure 4a), reaching values comparable to those of CTR + veh and CTR + MLT mice. Furthermore, treating BTBR mice with MLT reduced Vh and slightly reduced Cd (Figure 4b,c). These changes had a positive impact on the Vh–Cd ratio (Figure 4d).

The morphological observations described above are supported by the semi-quantitative analyses of inflammatory cells (Table 2), the morphometric analysis of the number of Pcs in the crypts (Figure 4a), and evaluations of Vh, Cd, and the Vh–Cd ratio (Figure 4b–d).

**Figure 5:**
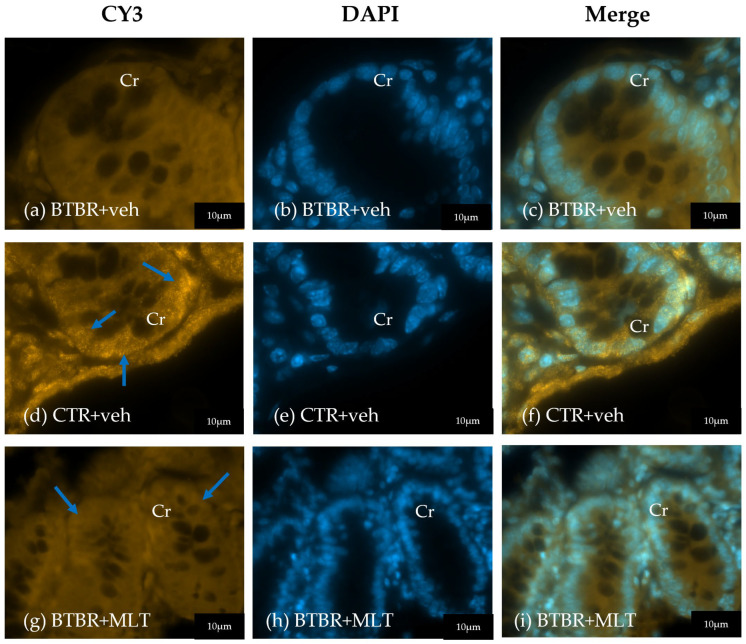
Representative photomicrographs of α-defensin 5 immunofluorescence staining of ileum samples of BTBR + veh (**a**–**c**), CTR + veh (**d**–**f**), and BTBR + MLT (**g**–**i**) mice. Cr: crypt. The blue arrows indicate immunopositivity. Scale bar: 20 µm/10 µm.

In addition to the count of Pcs, α-defensin 5 expression was also evaluated. In CTR + veh mice, α-defensin 5 immunopositivity was localized in the Pcs with a moderate/strong immunopositivity at cytoplasm granules (Figure 5a–c). In contrast, BTBR + veh mice exhibited fewer Pcs with very weak/absence of α-defensin 5 immunopositivity (Figure 5d–f). BTBR + MLT mice presented a slight increase in Pcs with a weak/very weak immunopositivity (Figure 5g–i).

### 3.3. Evaluation of Tight Junctions in the Ileum

#### 3.3.1. Immunohistochemical and Immunomorphometric Analyses of Claudin-1

**Figure 6:**
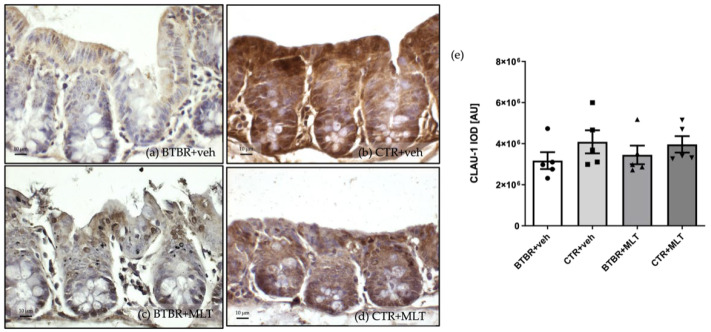
Representative photomicrographs of claudin-1 immunostaining of ileum samples of BTBR + veh (**a**), CTR + veh (**b**), BTBR + MLT (**c**), and CTR + MLT (**d**) mice. The graph (**e**) summarizes the immunomorphometric analysis of claudin-1. Ordinary one-way ANOVA with Šídák’s multiple-comparison test; F (3; 16) = 0.8726; *p*-value = 0.4757. BTBR + veh (*n* = 5) vs. CTR + veh (*n* = 5): mean ± S.E.M. = 3,173,949 ± 411,012 vs. 4,087,655 ± 561,716; *p*-value = 0.4476. BTBR + veh (*n* = 5) vs. BTBR + MLT (*n* = 5): mean ± S.E.M. = 3,173,949 ± 411,012 vs. 3,450,755 ± 451,906; *p*-value = 0.9661. CTR + veh (*n* = 5) vs. CTR + MLT (*n* = 5): mean ± S.E.M. = 4,087,655 ± 561,716 vs. 3,963,268 ± 398,339; *p*-value = 0.9967. AU: arbitrary unit; CLAU-1: claudin-1. Scale bar: 10 µm.

Claudin-1 immunopositivity was localized in the cytoplasm of enterocytes and Pcs in the different experimental groups (Figure 6). In these cells, claudin-1 immunopositivity was weak/moderate in BTBR + veh mice (Figure 6a) compared to CTR + veh and CTR + MLT mice, which showed strong immunopositivity (Figure 6b,d). Notably, MLT administration in BTBR mice induced a moderate increase in claudin-1 immunopositivity (Figure 6c).

These results are supported by the immunomorphometric analysis of claudin-1 immunopositivity, as summarized in Figure 6e.

#### 3.3.2. Immunohistochemical and Immunomorphometric Analyses of Claudin-2

**Figure 7:**
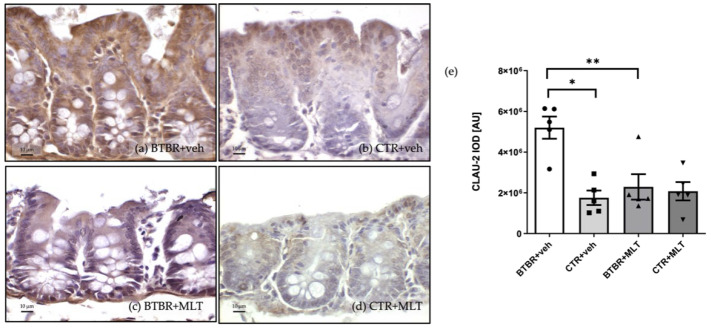
Representative photomicrographs of claudin-2 immunostaining of ileum samples from BTBR + veh (**a**), CTR + veh (**b**), BTBR + MLT (**c**), and CTR + MLT (**d**) mice. The graph (**e**) summarizes the immunomorphometric analyses of claudin-2. Ordinary one-way ANOVA with Šídák’s multiple-comparison test; F (3; 16) = 10.01; *p*-value = 0.0006. BTBR + veh (*n* = 5) vs. CTR + veh (*n* = 5): mean ± S.E.M. = 5,200,718 ± 544,355 vs. 1,758,436 ± 355,854; *p*-value = 0.0006 (*). BTBR + veh (*n* = 5) vs. BTBR + MLT (*n* = 5): mean ± S.E.M. = 5,200,718 ± 544,355 vs. 2,291,542 ± 623,232; *p*-value = 0.0026 (**). CTR + veh (*n* = 5) vs. CTR + MLT (*n* = 5): mean ± S.E.M. = 1,758,436 ± 355,854 vs. 2,079,988 ± 451,148; *p*-value = 0.9599. AU: arbitrary unit; CLAU-2: claudin-2. Scale bar: 10 µm.

Similarly to claudin-1, claudin-2 immunopositivity was observed in the cytoplasm of the enterocytes and Pcs in the different experimental groups (Figure 7). Claudin-2 expression was moderate/strong in BTBR + veh mice (Figure 7a), while CTR + veh and CTR + MLT mice showed very weak/weak expression (Figure 7b,d). Notably, MLT administration in BTBR mice reduced claudin-2 expression from strong to moderate/weak (Figure 7c).

The above observations are confirmed by the immunomorphometric analyses of claudin-2 immunopositivity, as summarized in Figure 7e. Specifically, claudin-2 immunopositivity was significantly higher in BTBR + veh mice than in CTR + veh mice (*p*-value < 0.001), and MLT administration in BTBR mice significantly reduced claudin-2 expression (*p*-value < 0.01).

#### 3.3.3. Immunohistochemical and Immunomorphometric Analyses of Zonula Occludens-1

**Figure 8:**
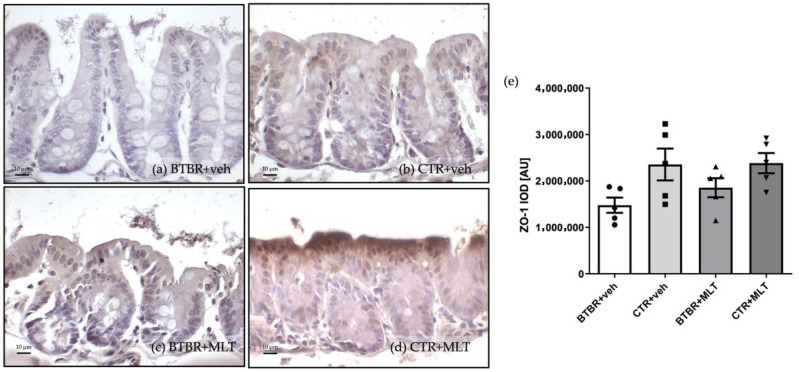
Representative photomicrographs of zonula occludens-1 immunostaining of ileum samples from BTBR + veh (**a**), CTR + veh (**b**), BTBR + MLT (**c**), and CTR + MLT (**d**) mice. The graph (**e**) summarizes the immunomorphometric analyses of zonula occludens-1. Ordinary one-way ANOVA with Šídák’s multiple-comparison test; F (3; 16) = 3.226; *p*-value = 0.0505. BTBR + veh (*n* = 5) vs. CTR + veh (*n* = 5): mean ± S.E.M. = 1,476,732 ± 164,507 vs. 2,353,744 ± 343,263; *p*-value = 0.0612. BTBR + veh (*n* = 5) vs. BTBR + MLT (*n* = 5): mean ± S.E.M. = 1,476,732 ± 164,507 vs. 1,853,473 ± 204,502; *p*-value = 0.6377. CTR + veh (*n* = 5) vs. CTR + MLT (*n* = 5): mean ± S.E.M. = 2,353,744 ± 343,263 vs. 2,383,553 ± 217,641; *p*-value = 0.9997. AU: arbitrary unit; ZO-1: zonula occludens-1. Scale bar: 10 µm.

ZO-1 immunopositivity was observed in the cytoplasm of enterocytes, located in the upper part of the villi, and in the cytoplasm of Pcs in the different experimental groups (Figure 8). Specifically, ZO-1 expression was negative/very weak in enterocytes and Pcs of BTBR + veh mice (Figure 8a) compared to CTR + veh and CTR + MLT mice (Figure 8b,d). MLT slightly increased ZO-1 expression in BTBR mice (Figure 8c).

These observations are supported by the immunomorphometric analyses of ZO-1 immunopositivity, as summarized in Figure 8e.

## 4. Discussion

This study demonstrates the promising beneficial effects of MLT in mitigating intestinal barrier impairment in a mouse model of ASD. Through morphological and morphometrical analyses, it was demonstrated that MLT improves the morphology of the small intestine and modulates the expression of key biomarkers involved in barrier integrity, such as claudins and ZO-1.

The exact etiology of ASD is not well understood, but it has been suggested that it involves the interaction between different signaling pathways that regulate the gut–brain axis. Previously, we reported that BTBR mice exhibit both brain and intestinal abnormalities [27]. Intestinal alterations cause changes in epithelial cell morphology and an increase in the number of inflammatory cells, disrupting the delicate balance of trafficking between the intestinal lumen and the submucosa [50].

Based on these findings, we focused our study on a defined part of the small intestine, the ileum of BTBR mice, to better characterize these alterations.

Firstly, we confirmed typical ASD behavior in BTBR + veh mice; this animal group spent more time performing self-grooming compared to CTR + veh and CTR + MLT mice. However, MLT administration improved this negative behavior in BTBR mice.

We evaluated the morphology of the ileum and the expression of proteins involved in barrier integrity, as described above. Our results confirm the increase in the number of inflammatory cells in BTBR mice, along with new findings regarding changes in intestinal Vh and Cd, as well as altered expression of three key TJs. The inflammatory state in BTBR mice likely resulted from disrupted intestinal barrier integrity, which triggers immune responses linked to conditions such as inflammatory bowel disorders and multiple sclerosis [51]. There is substantial evidence that intestinal inflammation alters BBB permeability and may contribute to the pathogenesis of ASD [2]. Furthermore, patients with ASD experience GI symptoms that may be associated with an impaired intestinal barrier. Several studies have highlighted the role of an incomplete intestinal barrier in the development and severity of ASD [4,52,53].

Interestingly, BTBR mice have longer intestinal villi, reduced Cd, and an increased Vh–Cd ratio compared to CTR mice. Our previous work documented longer intestinal villi in another part of the small intestine (jejunum) of BTBR mice [27], suggesting that Vh alterations affect exchanges between the intestinal mucosa and luminal contents, which may alter microbiota composition, interfering with oxidative stress and apoptosis pathways.

Enterocytes, which make up the majority of villus cells, are the primary absorptive cells in the small intestine [54]. An increase in the absorption surface may lead to improved nutrient transport on the villus surface [55], potentially contributing to the increased body weight. Increased body weight has been observed in this and previous studies on BTBR mice [49].

In the present study, we also analyzed the Vh–Cd ratio and found that it was higher in BTBR mice compared to CTR mice. We observed an increase in Vh and a decrease in Cd in BTBR mice, while the opposite pattern occurred in CTR animals, supporting an inverse correlation between these parameters [56]. Furthermore, the number of Pcs, identified morphologically in the intestinal crypts, was significantly reduced in BTBR mice. Pcs are specialized secretory cells that produce antimicrobial peptides essential for host defense and the maintenance of the intestinal microbiota [24,25]. Furthermore, in the present study, we observed that BTBR + veh mice presented few Pcs with absent α-defensin 5 immunopositivity compared to CTR + veh mice in which α-defensin 5 was moderately present in Pcs cytoplasm granules. Notably, MLT administration in BTBR mice promoted a slight increase in α-defensin 5 in Pcs. Multiple lines of evidence suggest that Pcs dysfunction and α-defensins reduced expression may increase susceptibility to gut alterations and diseases [57,58,59]. Due to the morphological barrier alteration observed in BTBR mice, we hypothesize that the reduced number of these cells in BTBR mice contributes to gut dysbiosis. Further studies are needed to confirm and expand this hypothesis.

To further investigate the role of the intestinal epithelium in regulating the traffic between the lumen and intracellular environment, we evaluated the TJs in this ASD model. The intestinal epithelium forms the longest mucosal barrier between the external environment and the body. This relationship is mediated by TJs, which control paracellular permeability [11].

TJs are emerging as therapeutic targets for GI diseases, so we analyzed their expression in BTBR mice. No differences in protein localization were observed between BTBR and CTR animals, while differences in TJs expression were detected. Claudin-1 expression was reduced, while claudin-2 expression was increased in BTBR mice compared to CTR animals, demonstrating opposing regulation. Previous loss-and gain-of-function studies highlight the roles of TJs barrier function and ion permeability in physiological and pathological conditions [60]. These findings support the idea that a compromised intestinal barrier allows the translocation of endotoxins into extraintestinal tissues, including the liver, portal circulation, lymph nodes, and nervous system [13].

We also examined ZO-1, a key regulator of TJ complexes [50], and we found that its expression was deregulated in BTBR mice compared to CTR animals, although its localization remained unchanged.

Consistently, the decreased expression of ZO-1 agrees with data reported by Qaisar et al. [61] on patients with Alzheimer’s disease. These authors suggested that, in these patients, the leakage of ZO-1 from the intestine into the circulation reinforces the hypothesis that ZO-1 is dysregulated in the intestine and upregulated in serum. This result is also consistent with previous research on intestinal permeability, which indicates a strong correlation between serum ZO-1 levels and the severity of GI symptoms in patients with ASD [20].

Taken together, these findings suggest the fundamental role of ZO-1 in contributing to the physiological regulation of the TJ complex in the small intestine and thus modulating the gut–brain axis in patients with ASD. The correlation between the ZO-1 expression and levels of symptom severity in patients with ASD indicates that this protein may serve as a biomarker for assessing intestinal dysfunction in neurodevelopmental disorders.

It is important to note that MLT treatment mitigates morphological alterations in the ileum and normalizes TJs expression in BTBR mice. The role of MLT has been extensively investigated in pathophysiological conditions, including ASD. Reduced MLT secretion has been observed in individuals with ASD, and its administration is a well-established and well-tolerated treatment for sleep disorders in children and adolescents with ASD [62,63]. In addition to regulating sleep, MLT has been shown to attenuate stress-induced injury, improve gut microbiota composition, and alleviate neurological and hepatic dysfunction in BTBR mice [37,39,64].

In conclusion, MLT’s protective effects on the intestinal barrier in BTBR mice may be partially attributed to its well-known anti-inflammatory and antioxidant properties [36,46,65,66,67,68]. Furthermore, we suggest that its beneficial impact on TJs may be related to MLT’s ability to act as a signaling molecule that modulates intestinal barrier morphology. MLT appears to increase the number of secretory cells and to modify the Vh in the intestinal epithelium.

Furthermore, it seems that BTBR mice presented increased corticosteroid levels, which are commonly related to a decrease in the serum level of MLT [40,69,70]. These mice probably presented an altered circadian rhythm, which influences the intestinal bacterial settlement. Mantani et al. [71] reported that the interaction between various intestinal cells and bacteria that settle on the gut mucosa can change diurnally in relation to circadian rhythm. This hypothesis led to the idea that circadian changes may affect villus metrics. Further studies on this topic are needed.

## 5. Conclusions

Our findings are consistent with previous results from an experimental study in which the authors focused on the effects of MLT on intestinal mucosal injury and microbiota dysbiosis in sleep-deprived mice. They suggested that MLT supplementation reversed dysbiosis and the inflammatory state in the colon [72]. Intestinal tissue contains many MLT receptors and absorbs a large amount of MLT, indicating that MLT plays an important role in regulating intestinal function [72,73].

MLT normalizes TJ expression, and histological indices are consistent with improved barrier function/morphology. These findings suggest the potential use of MLT as a co-therapeutic agent for intestinal disorders, including leaky gut. Future studies should focus on elucidating the molecular mechanisms underlying MLT’s effects to optimize its therapeutic application. The low price of MLT and its high safety margin also suggest broad possibilities for the use of this oral supplementary compound.

## 6. Limitations and Future Perspective

The results of this study should be interpreted cautiously due to its limitations of a small sample size and only male mice, together with the absence of microbiota or functional permeability analyses. Furthermore, it is known that sex and age affect gut permeability and microbiota [74,75,76]. Women, particularly young adult women, display greater gut microbial diversity than males [75]. Furthermore, female mice have higher baseline intestinal permeability, and the expression of genes involved in mucus biosynthesis in the ileum is differentially regulated in old male mice compared to old female mice [77,78]. There is conflicting evidence on whether there are differences between males and females in circadian timing [79,80]. However, females mice exhibit a nocturnal MLT peak which is 38–41% higher than that of males [81,82]. Nevertheless, the suppression of MLT production due to exposure to bright light does not appear to be influenced differently by sex [83,84]. Future work should integrate barrier function analyses, microbiome/metabolome, behavioral rescue, sex differences, and the timing and dose of MLT treatment. Peters et al. [85] reported that MLT receptor activation in the intestine may be a potent candidate for the oral treatment of a compromised intestinal mucosal barrier. Therefore, the MLT receptor mechanism(s) will be investigated using the high-affinity MLT receptor antagonist luzindole (N-acetyl-2-benzyltryptamine) [86] to determine whether the protective actions of MLT are receptor-mediated or if other pathways not influenced by MLT receptors are activated.

## Figures and Tables

**Table 1:** Rodent diet composition and macronutrient data.

Composition	Calories from Protein	Calories from Fat	Calories from Carbohydrate	Analytical Constituents	%
Wheat, maize, extracted and toasted soybean meal, corn gluten feed, wheat straw, fish meal, lucerne meal, mineral dicalcium phosphate, calcium carbonate, sodium chloride, whey powder, soybean oil, yeasts.	24%	18%	58%	Moisture	12.00
				Crude protein	18.50
				Crude oils and fats	3.00
				Crude fibers	6.00
				Crude ash	7.00

**Table 2:** The table summarizes the semi-quantitative analysis of inflammatory cells in the submucosa of BTBR + veh (*n* = 3), CTR + veh (*n* = 2), BTBR + MLT (*n* = 2), and CTR + MLT (*n* = 2). The number of inflammatory cells is expressed as absent (−), very weak (+/−), weak (+), moderate (++), and strong (+++).

Experimental Group	BTBR + veh	CTR + veh	BTBR + MLT	CTR + MLT
Semi-quantitative evaluation of inflammatory cells	++	+/++	+/++	+/−/+

## Data Availability

The data supporting this article will be shared upon reasonable request to the corresponding author.

## Supplementary Materials

The following supporting information can be downloaded at https://www.mdpi.com/article/10.3390/biology14111594/s1. Figure S1: Body weight progression of all experimental groups monitored at three time points over 16 weeks (week 4, week 12, and week 21); Table S1: Time spent performing self-grooming (in seconds) during the 10 min observation test.

## References

[1] Cortese S., Bellato A., Gabellone A., Marzulli L., Matera E., Parlatini V., Petruzzelli M.G., Persico A.M., Delorme R., Fusar-Poli P. (2025). Latest Clinical Frontiers Related to Autism Diagnostic Strategies. Cell Rep. Med..

[2] Rowshan N., Anjomshoa M., Farahzad A., Bijad E., Amini-Khoei H. (2024). Gut-Brain Barrier Dysfunction Bridge Autistic-like Behavior in Mouse Model of Maternal Separation Stress: A Behavioral, Histopathological, and Molecular Study. Int. J. Dev. Neurosci..

[3] Sauer A.K., Stanton J.E., Hans S., Grabrucker A.M., Grabrucker A.M. (2021). Autism Spectrum Disorders: Etiology and Pathology. Autism Spectrum Disorders.

[4] Longo B., Andriolo I.R.L., de Melo D.M., de Souza M.M., Prediger R.D., da Silva L.M. (2025). Gastrointestinal Manifestations in Autism Spectrum and Attention-Deficit/Hyperactivity Disorders: Pathogenesis and Drug Targets. Curr. Dev. Disord. Rep..

[5] Bi D., Huang J., Zhu N., Yao L., Wu Y., Peng Y., Chen G., Zhu B., Xu X. (2025). Elucidation of Molecular Mechanisms of Sulfated Oligoguluronic Acid on Mitigating Intestinal Inflammation and Enhancing Epithelial Barrier Function. J. Agric. Food Chem..

[6] Li B., Hsieh Y.-R., Lai W.-D., Tung T.-H., Chen Y.-X., Yang C.-H., Fang Y.-C., Huang S.-Y. (2023). Melatonin Ameliorates Neuropsychiatric Behaviors, Gut Microbiome, and Microbiota-Derived Metabolites in Rats with Chronic Sleep Deprivation. Int. J. Mol. Sci..

[7] Ristori M.V., Quagliariello A., Reddel S., Ianiro G., Vicari S., Gasbarrini A., Putignani L. (2019). Autism, Gastrointestinal Symptoms and Modulation of Gut Microbiota by Nutritional Interventions. Nutrients.

[8] Moonwiriyakit A., Pathomthongtaweechai N., Steinhagen P.R., Chantawichitwong P., Satianrapapong W., Pongkorpsakol P. (2023). Tight Junctions: From Molecules to Gastrointestinal Diseases. Tissue Barriers.

[9] Shindler A.E., Hill-Yardin E.L., Petrovski S., Cunningham A.C., Bishop N., Franks A.E. (2020). Potential Determinants of Gastrointestinal Dysfunction in Autism Spectrum Disorders. Rev. J. Autism Dev. Disord..

[10] Suzuki T. (2013). Regulation of Intestinal Epithelial Permeability by Tight Junctions. Cell. Mol. Life Sci..

[11] Suzuki T. (2020). Regulation of the Intestinal Barrier by Nutrients: The Role of Tight Junctions. Anim. Sci. J..

[12] Turner J.R. (2009). Intestinal Mucosal Barrier Function in Health and Disease. Nat. Rev. Immunol..

[13] Assimakopoulos S.F., Papageorgiou I., Charonis A. (2011). Enterocytes’ Tight Junctions: From Molecules to Diseases. World J. Gastrointest. Pathophysiol..

[14] Al-Ayadhi L., Zayed N., Bhat R.S., Moubayed N.M.S., Al-Muammar M.N., El-Ansary A. (2021). The Use of Biomarkers Associated with Leaky Gut as a Diagnostic Tool for Early Intervention in Autism Spectrum Disorder: A Systematic Review. Gut Pathog..

[15] Caviglia G.P., Rosso C., Ribaldone D.G., Dughera F., Fagoonee S., Astegiano M., Pellicano R. (2019). Physiopathology of Intestinal Barrier and the Role of Zonulin. Minerva Biotecnol..

[16] Veres-Székely A., Szász C., Pap D., Szebeni B., Bokrossy P., Vannay Á. (2023). Zonulin as a Potential Therapeutic Target in Microbiota-Gut-Brain Axis Disorders: Encouraging Results and Emerging Questions. Int. J. Mol. Sci..

[17] Fasano A. (2008). Physiological, Pathological, and Therapeutic Implications of Zonulin-Mediated Intestinal Barrier Modulation. Am. J. Pathol..

[18] Tirelli E., Pucci M., Squillario M., Bignotti G., Messali S., Zini S., Bugatti M., Cadei M., Memo M., Caruso A. (2025). Effects of Methylglyoxal on Intestine and Microbiome Composition in Aged Mice. Food Chem. Toxicol..

[19] Fasano A. (2011). Zonulin and Its Regulation of Intestinal Barrier Function: The Biological Door to Inflammation, Autoimmunity, and Cancer. Physiol. Rev..

[20] Sonbol H.M., Abdelmawgoud A.S., El-kady N.M., Abdelhay E.S., Abdel Tawab H.E. (2025). Serum Zonulin Level in Autistic Children and Its Relation to Severity of Symptoms a Case-Control Study. Sci. Rep..

[21] Wang J., Zheng B., Zhou D., Xing J., Li H., Li J., Zhang Z., Zhang B., Li P. (2020). Supplementation of Diet with Different N-3/n-6 PUFA Ratios Ameliorates Autistic Behavior, Reduces Serotonin, and Improves Intestinal Barrier Impairments in a Valproic Acid Rat Model of Autism. Front. Psychiatry.

[22] Luettig J., Rosenthal R., Barmeyer C., Schulzke J.D. (2015). Claudin-2 as a Mediator of Leaky Gut Barrier during Intestinal Inflammation. Tissue Barriers.

[23] Ehmann D., Wendler J., Koeninger L., Larsen I.S., Klag T., Berger J., Marette A., Schaller M., Stange E.F., Malek N.P. (2019). Paneth Cell α-Defensins HD-5 and HD-6 Display Differential Degradation into Active Antimicrobial Fragments. Proc. Natl. Acad. Sci. USA.

[24] Nakamura K., Sakuragi N., Takakuwa A., Ayabe T. (2016). Paneth Cell α-Defensins and Enteric Microbiota in Health and Disease. Biosci. Microbiota Food Health.

[25] Bevins C.L., Salzman N.H. (2011). Paneth Cells, Antimicrobial Peptides and Maintenance of Intestinal Homeostasis. Nat. Rev. Microbiol..

[26] Petropoulos A., Stavropoulou E., Tsigalou C., Bezirtzoglou E. (2025). Microbiota Gut-Brain Axis and Autism Spectrum Disorder: Mechanisms and Therapeutic Perspectives. Nutrients.

[27] Franco C., Gianò M., Favero G., Rezzani R. (2022). Impairment in the Intestinal Morphology and in the Immunopositivity of Toll-like Receptor-4 and Other Proteins in an Autistic Mouse Model. Int. J. Mol. Sci..

[28] Meyza K.Z., Blanchard D.C. (2017). The BTBR Mouse Model of Idiopathic Autism–Current View on Mechanisms. Neurosci. Biobehav. Rev..

[29] Tan D.X., Xu B., Zhou X., Reiter R.J. (2018). Pineal Calcification, Melatonin Production, Aging, Associated Health Consequences and Rejuvenation of the Pineal Gland. Molecules.

[30] Yasmin F., Sutradhar S., Das P., Mukherjee S. (2021). Gut Melatonin: A Potent Candidate in the Diversified Journey of Melatonin Research. Gen. Comp. Endocrinol..

[31] Pereira N., Naufel M.F., Ribeiro E.B., Tufik S., Hachul H. (2020). Influence of Dietary Sources of Melatonin on Sleep Quality: A Review. J. Food Sci..

[32] Alshehri F.S., Alghamdi B.S., Hakami A.Y., Alshehri A.A., Althobaiti Y.S. (2021). Melatonin Attenuates Morphine-Induced Conditioned Place Preference in Wistar Rats. Brain Behav..

[33] Favero G., Franceschetti L., Bonomini F., Rodella L.F., Rezzani R. (2017). Melatonin as an Anti-Inflammatory Agent Modulating Inflammasome Activation. Int. J. Endocrinol..

[34] Guo R., Rao P., Liao B., Luo X., Yang W., Lei X., Ye J. (2025). Melatonin Suppresses PD-L1 Expression and Exerts Antitumor Activity in Hepatocellular Carcinoma. Sci. Rep..

[35] Mohammadi N., Alizadeh M., Akbarzadeh S., Rezaei M., Mahmoodi M., Netticadan T., Movahed A. (2025). Melatonin Administered Postoperatively Lowers Oxidative Stress and Inflammation and Significantly Recovers Heart Function in Patients Undergoing CABG Surgery. Eur. J. Med. Res..

[36] Reiter R.J., Mayo J.C., Tan D.-X., Sainz R.M., Alatorre-Jimenez M., Qin L. (2016). Melatonin as an Antioxidant: Under Promises but over Delivers. J. Pineal Res..

[37] Lin R., Wang Z., Cao J., Gao T., Dong Y., Chen Y. (2020). Role of Melatonin in Intestinal Mucosal Injury Induced by Restraint Stress in Mice. Pharm. Biol..

[38] Sommansson A., Yamskova O., Schiöth H.B., Nylander O., Sjöblom M. (2014). Long-Term Oral Melatonin Administration Reduces Ethanol-Induced Increases in Duodenal Mucosal Permeability and Motility in Rats. Acta Physiol..

[39] Rezzani R., Gianò M., Pinto D., Rinaldi F., van Noorden C.J.F., Favero G. (2024). Hepatic Alterations in a BTBR T + Itpr3tf/J Mouse Model of Autism and Improvement Using Melatonin via Mitigation Oxidative Stress, Inflammation and Ferroptosis. Int. J. Mol. Sci..

[40] Borsani E., Bonomini F., Bonini S.A., Premoli M., Maccarinelli G., Giugno L., Mastinu A., Aria F., Memo M., Rezzani R. (2022). Role of Melatonin in Autism Spectrum Disorders in a Male Murine Transgenic Model: Study in the Prefrontal Cortex. J. Neurosci. Res..

[41] Adiguzel C., Karaboduk H., Uzunhisarcikli M. (2024). Protective Role of Melatonin Against Abamectin-Induced Biochemical, Immunohistochemical, and Ultrastructural Alterations in the Testicular Tissues of Rats. Microanal.

[42] Dang J., Yu Z., Wang T., Jiao Y., Wang K., Dou W., Yi C., Song B. (2023). Effects of Melatonin on Fat Graft Retention Through Browning of Adipose Tissue and Alternative Macrophage Polarization. Aesthet. Plast. Surg..

[43] Ghorbani F., Osatd-Rahimi N., Mansouritorghabeh F., Ebrahimzadeh-Bideskan A., Saburi E., Rajabian A., Hosseini M. (2025). Methamphetamine Exposure during Gestation and Lactation Periods Impairs the Learning and Memory of Offspring Mice, Which Is Reversed by Melatonin: The Role of Oxidative Stress and Acetylcholinesterase. Res. Pharm. Sci..

[44] Andersen L.P.H., Werner M.U., Rosenkilde M.M., Harpsøe N.G., Fuglsang H., Rosenberg J., Gögenur I. (2016). Pharmacokinetics of Oral and Intravenous Melatonin in Healthy Volunteers. BMC Pharmacol. Toxicol..

[45] Silverman J.L., Tolu S.S., Barkan C.L., Crawley J.N. (2010). Repetitive Self-Grooming Behavior in the BTBR Mouse Model of Autism Is Blocked by the mGluR5 Antagonist MPEP. Neuropsychopharmacology.

[46] Favero G., Gianò M., Franco C., Pinto D., van Noorden C.J.F., Rinaldi F., Rezzani R. (2024). Relation Between Reactive Oxygen Species Production and Transient Receptor Potential Vanilloid1 Expression in Human Skin During Aging. J. Histochem. Cytochem..

[47] Chieco P., Jonker A., De Boer B.A., Ruijter J.M., Van Noorden C.J.F. (2013). Image Cytometry: Protocols for 2D and 3D Quantification in Microscopic Images. Prog. Histochem. Cytochem..

[48] Wiseman E.J., Moss J.I., Atkinson J., Baakza H., Hayes E., Willis S.E., Waring P.M., Rodriguez Canales J., Jones G.N. (2023). Epitope Lability of Phosphorylated Biomarkers of the DNA Damage Response Pathway Results in Increased Vulnerability to Effects of Delayed or Incomplete Formalin Fixation. J. Histochem. Cytochem..

[49] Franco C., Bonomini F., Borsani E., Castrezzati S., Franceschetti L., Rezzani R. (2021). Involvement of Intestinal Goblet Cells and Changes in Sodium Glucose Transporters Expression: Possible Therapeutic Targets in Autistic BTBR T^+^Itpr3^tf^/J Mice. Int. J. Environ. Res. Public Health.

[50] Sturgeon C., Fasano A. (2016). Zonulin, a Regulator of Epithelial and Endothelial Barrier Functions, and Its Involvement in Chronic Inflammatory Diseases. Tissue Barriers.

[51] Nalbant K., Erden S., Yazar A., Kılınç İ. (2022). Investigation of the Relation between Epithelial Barrier Function and Autism Symptom Severity in Children with Autism Spectrum Disorder. J. Mol. Neurosci..

[52] Dargenio V.N., Dargenio C., Castellaneta S., De Giacomo A., Laguardia M., Schettini F., Francavilla R., Cristofori F. (2023). Intestinal Barrier Dysfunction and Microbiota–Gut–Brain Axis: Possible Implications in the Pathogenesis and Treatment of Autism Spectrum Disorder. Nutrients.

[53] Teskey G., Anagnostou E., Mankad D., Smile S., Roberts W., Brian J., Bowdish D.M.E., Foster J.A. (2021). Intestinal Permeability Correlates with Behavioural Severity in Very Young Children with ASD: A Preliminary Study. J. Neuroimmunol..

[54] Gerbe F., Legraverend C., Jay P. (2012). The Intestinal Epithelium Tuft Cells: Specification and Function. Cell. Mol. Life Sci..

[55] Seyyedin S., Nazem M.N. (2017). Histomorphometric Study of the Effect of Methionine on Small Intestine Parameters in Rat: An Applied Histologic Study. Folia Morphol..

[56] Daveson A.J.M., Popp A., Taavela J., Goldstein K.E., Isola J., Truitt K.E., Mäki M., Anderson R.P., Adams A., The RESET CeD Study Group (2020). Baseline Quantitative Histology in Therapeutics Trials Reveals Villus Atrophy in Most Patients with Coeliac Disease Who Appear Well Controlled on Gluten-free Diet. GastroHep.

[57] Adolph T.E., Tomczak M.F., Niederreiter L., Ko H.-J., Böck J., Martinez-Naves E., Glickman J.N., Tschurtschenthaler M., Hartwig J., Hosomi S. (2013). Paneth Cells as a Site of Origin for Intestinal Inflammation. Nature.

[58] Stappenbeck T.S., McGovern D.P.B. (2017). Paneth Cell Alterations in the Development and Phenotype of Crohn’s Disease. Gastroenterology.

[59] Wehkamp J., Stange E.F. (2020). An Update Review on the Paneth Cell as Key to Ileal Crohn’s Disease. Front. Immunol..

[60] Zeisel M.B., Dhawan P., Baumert T.F. (2019). Tight Junction Proteins in Gastrointestinal and Liver Disease. Gut.

[61] Qaisar R., Karim A., Iqbal M.S., Ahmad F., Shaikh A., Kamli H., Khamjan N.A. (2023). A Leaky Gut Contributes to Postural Dysfunction in Patients with Alzheimer’s Disease. Heliyon.

[62] Ding W., Xu Y., Ding W., Tang Q., Zhang B., Yuan Y., Jin J. (2025). Research Progress on Melatonin, 5-HT, and Orexin in Sleep Disorders of Children with Autism Spectrum Disorder. Biomol. Biomed..

[63] Lalanne S., Fougerou-Leurent C., Anderson G.M., Schroder C.M., Nir T., Chokron S., Delorme R., Claustrat B., Bellissant E., Kermarrec S. (2021). Melatonin: From Pharmacokinetics to Clinical Use in Autism Spectrum Disorder. Int. J. Mol. Sci..

[64] Bonetti M., Giugno L., Borsani E., Bonomini F. (2024). Potential Neuroprotective Effect of Melatonin in the Hippocampus of Male BTBR Mice. Nutrients.

[65] Hussein E.M., Ghanem N.F., Bakr S.M., Kasem S.M., Dkhil M.A., Thagfan F.A., Essawy A.E. (2025). Microscopic and Ultrastructural Insights into the Protective Role of Melatonin against Tartrazine-Induced Hepatotoxicity. Biotech. Histochem..

[66] Joo S.S., Yoo Y.-M. (2025). Protective Effect of Melatonin Against Bisphenol A Toxicity. Int. J. Mol. Sci..

[67] Moretti R., Zanin A., Pansiot J., Spiri D., Manganozzi L., Kratzer I., Favero G., Vasiljevic A., Rinaldi V.E., Pic I. (2015). Melatonin Reduces Excitotoxic Blood–Brain Barrier Breakdown in Neonatal Rats. Neuroscience.

[68] Sun Z., Chai L., Li D., Yang Y., Yao W., Li H., Shan C., Wen X., Lin R. (2025). Role of Melatonin in Intestinal Mucosal Injury Induced by Chronic Restraint Stress in Mice. Neuroendocrinology.

[69] Dokoohaki S., Ghareghani M., Ghanbari A., Farhadi N., Zibara K., Sadeghi H. (2017). Corticosteroid Therapy Exacerbates the Reduction of Melatonin in Multiple Sclerosis. Steroids.

[70] Frye R.E., James S.J. (2014). Metabolic Pathology of Autism in Relation to Redox Metabolism. Biomark. Med..

[71] Mantani Y., Sakata N., Kubota N., Shimada A., Nakanishi S., Yokoyama T., Hoshi N. (2023). Diurnal Changes in Bacterial Settlement on the Peyer’s Patch and Surrounding Mucosa in the Rat Ileum and Its Effect against the Intestinal Immune System. Cell Tissue Res..

[72] Gao T., Wang Z., Dong Y., Cao J., Lin R., Wang X., Yu Z., Chen Y. (2019). Role of Melatonin in Sleep Deprivation-Induced Intestinal Barrier Dysfunction in Mice. J. Pineal Res..

[73] Kromm F., Baumann A., Sánchez V., Brandt A., Staltner R., Bergheim I. (2025). Oral Supplementation of Melatonin Attenuates the Onset of Alcohol-Related Liver Disease. J. Mol. Med..

[74] El-Hakim Y., Mani K.K., Eldouh A., Pandey S., Grimaldo M.T., Dabney A., Pilla R., Sohrabji F. (2021). Sex Differences in Stroke Outcome Correspond to Rapid and Severe Changes in Gut Permeability in Adult Sprague-Dawley Rats. Biol. Sex Differ..

[75] Sharma A., Kapur S., Kancharla P., Yang T. (2025). Sex Differences in Gut Microbiota, Hypertension, and Cardiovascular Risk. Eur. J. Pharmacol..

[76] Rosser E.C., De Gruijter N.M., Matei D.E. (2022). Mini-Review: Gut-Microbiota and the Sex-Bias in Autoimmunity–Lessons Learnt from Animal Models. Front. Med..

[77] Elderman M., Sovran B., Hugenholtz F., Graversen K., Huijskes M., Houtsma E., Belzer C., Boekschoten M., de Vos P., Dekker J. (2017). The Effect of Age on the Intestinal Mucus Thickness, Microbiota Composition and Immunity in Relation to Sex in Mice. PLoS ONE.

[78] Volynets V., Reichold A., Bárdos G., Rings A., Bleich A., Bischoff S.C. (2016). Assessment of the Intestinal Barrier with Five Different Permeability Tests in Healthy C57BL/6J and BALB/cJ Mice. Dig. Dis. Sci..

[79] Gunn P.J., Middleton B., Davies S.K., Revell V.L., Skene D.J. (2016). Sex Differences in the Circadian Profiles of Melatonin and Cortisol in Plasma and Urine Matrices under Constant Routine Conditions. Chronobiol. Int..

[80] Lok R., Qian J., Chellappa S.L. (2024). Sex Differences in Sleep, Circadian Rhythms, and Metabolism: Implications for Precision Medicine. Sleep Med. Rev..

[81] Cain S.W., Dennison C.F., Zeitzer J.M., Guzik A.M., Khalsa S.B.S., Santhi N., Schoen M.W., Czeisler C.A., Duffy J.F. (2010). Sex Differences in Phase Angle of Entrainment and Melatonin Amplitude in Humans. J. Biol. Rhythm..

[82] Santhi N., Lazar A.S., McCabe P.J., Lo J.C., Groeger J.A., Dijk D.-J. (2016). Sex Differences in the Circadian Regulation of Sleep and Waking Cognition in Humans. Proc. Natl. Acad. Sci. USA.

[83] Nathan P.J., Burrows G.D., Norman T.R. (1997). The Effect of Dim Light on Suppression of Nocturnal Melatonin in Healthy Women and Men. J. Neural Transm..

[84] Nathan P.J., Wyndham E.L., Burrows G.D., Norman T.R. (2000). The Effect of Gender on the Melatonin Suppression by Light: A Dose Response Relationship. J. Neural Transm..

[85] Peters K., Dahlgren D., Lennernäs H., Sjöblom M. (2021). Melatonin-Activated Receptor Signaling Pathways Mediate Protective Effects on Surfactant-Induced Increase in Jejunal Mucosal Permeability in Rats. Int. J. Mol. Sci..

[86] Rezzani R., Rodella L.F., Bonomini F., Tengattini S., Bianchi R., Reiter R.J. (2006). Beneficial Effects of Melatonin in Protecting against Cyclosporine A-induced Cardiotoxicity Are Receptor Mediated. J. Pineal Res..

