# Supplementary material for: Melatonin Improves Intestinal Barrier Impairment in a Mouse Model of Autism Spectrum Disorder
Source: Biology (Basel). 2025 Nov 14;14(11):1594. doi: 10.3390/biology14111594 (PMC12650372; doi:10.3390/biology14111594)
Supplement: Supplementary file 1 [file biology-14-01594-s001.zip › biology-3905602-supplementary.pdf]

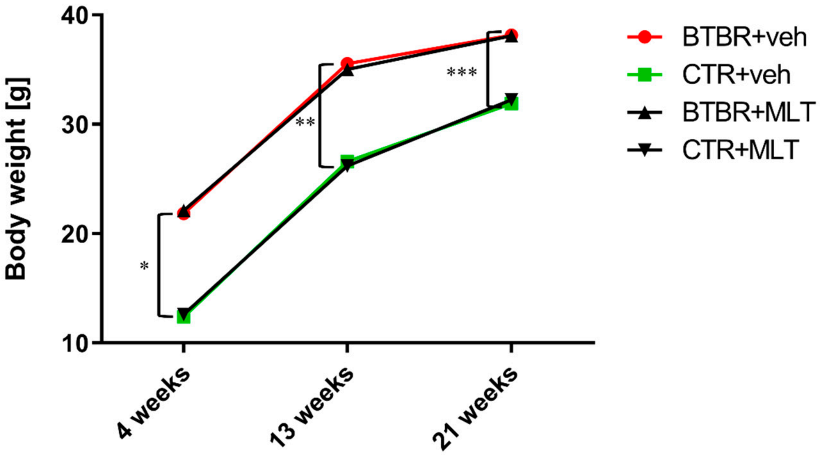

Figure S1. Body weight progression of all experimental groups monitored at three time points for 16 weeks (week 4, week 12, and week 21). Two-Way ANOVA with Tukey's multiple comparisons test. 4 weeks: BTBR+veh (n=10) vs. CTR+veh (n=10): mean ± S.E.M. = 21.84 ± 0.128 vs. 12.39 ± 0.124; p-value < 0.0001 (*). BTBR+veh (n=10) vs. BTBR+MLT (n=10): mean ± S.E.M. = 21.84 ± 0.128 vs. 22.11 ± 0.152; p-value = 0.6523. CTR+veh (n=10) vs. CTR+MLT (n=10): mean ± S.E.M. = 12.39 ± 0.124 vs. 12.62 ± 0.154; p-value = 0.7628. 13 weeks: BTBR+veh (n=10) vs. CTR+veh (n=10): mean ± S.E.M. = 35.55 ± 0.173 vs. 26.59 ± 0.209; p-value < 0.0001 (**). BTBR+veh (n=10) vs. BTBR+MLT (n=10): mean ± S.E.M. = 35.55 ± 0.173 vs. 35.01 ± 0.140; p-value = 0.1109. CTR+veh (n=10) vs. CTR+MLT (n=10): mean ± S.E.M. = 26.59 ± 0.209 vs. 26.20 ± 0.132; p-value = 0.3518. 21 weeks: BTBR+veh (n=10) vs. CTR+veh (n=10): mean ± S.E.M. = 38.14 ± 0.213 vs. 31.90 ± 0.239; p-value = < 0.0001 (***). BTBR+veh (n=10) vs. BTBR+MLT (n=10): mean ± S.E.M. = 38.14 ± 0.213 vs. 38.09 ± 0.163; p-value = 0.9963. CTR+veh (n=10) vs. CTR+MLT (n=10): mean ± S.E.M. = 31.90 ± 0.329 vs. 32.23 ± 0.152; p-value = 0.4965.

	BTBR+veh	CTR+veh	BTBR+MLT	CTR+MLT
Mean (s)	60,00	33,44	18,80	46,22
S.E.M.	11,66	4,51	3,89	4,46
p-value	0,0249 vs CTR+veh	/	0,0002 vs BTBR+veh	/

Table S1: Time spent doing self-grooming (in seconds) during the 10-minute observation test. The p-values of statistically significant results were reported in the table. One-way ANOVA followed by Tukey's multiple comparisons test was applied.
